# Complimentary Dinner to Dr. S. D. Gross

**Published:** 1879-05

**Authors:** 


### The Complimentary Dinner to Dr. S. D. Gross.

Philadelphia, April 12, 1879.

The complimentary dinner tendered to Prof. Samuel D. Gross, in commemoration of his fifty-first year in the practice of surgery, took place Tuesday evening, April 10th, at St. George's Hotel, southwest corner of Broad and Walnut streets, Philadelphia. The original plan had been that the dinner should be held at the Union League Club, but the accommodations not proving sufficient for the number of guests expected, the location was changed to the St. George's Hotel. The large dining-room of the hotel was richly decorated with plants and flowers. The music was furnished by Carl Sentz's orchestra.

The subscriptions to the dinner ($10 each) were limited to one hundred of Prof. Gross's professional friends in Philadelphia.

The congratulatory address to Dr. Gross. the first event on the programme, was delivered by Dr. Agnew, who, as he sat down, touched Dr. Gross on the shoulder and said: "Allow me, in the name of your professional friends, to pin this token on the lappel of your coat. It was a gold medal, set with diamonds and brilliants, and bearing on its reverse side this inscription:

"Presented to Dr. S. D. Gross by his medical friends in commemoration of his fifty-first year in the profession, April 10, 1879."

Dr. Gross responded as follows:

"In rising to the toast offered by the distinguished chairman, I feel deeply oppressed by what Dr. Rush has so well described as "suffocated excitement." You need not be assured how much I appreciate the honor conferred by the occasion and by this warm reception. The sentiments embodied in the toast touch my heart, and I should indeed be dead to all the finer feelings of my nature if I did not tender you my most cordial and respectful acknowledgments. It is no light compliment to be in such a presence, or to be the guest of such a company. To merit the approbation of my professional brethren and of good men generally has ever been my highest ambition, as it must be of every honest and virtuous citizen. The offer of a public dinner extended to me a few weeks ago by a committee of my professional friends, took me completely by surprise, and would probably have been promptly declined if it had not been accompanied by such kind and flattering words as at once to satisfy me that they came from the heart. The commendations which you have bestowed upon my private character and public services as a practitioner and teacher of surgery are measured, I am conscious, rather by your own generous feelings than by any deserts of mine. Whatever value those services may possess, it is no ordinary consolation to me to know that they are appreciated by men among whom I have lived for nearly a quarter of a century, with many of whom I have been brought into frequent contact in various relations of life—often, indeed, under circumstances of a most trying kind—with some of whom I have been officially associated, and with none of whom, thanks be to God, I have ever had one word of misunderstanding.

"It is not a pleasant thing to speak of oneself, but there are a few circumstances in the history of my uneventful life to which I may perhaps be pardoned for referring upon this occasion. I have grown old in the profession, as pupil and practitioner for fifty-four years, my graduation dating back to March, 1828. A little over a month ago I closed my thirty-ninth course of lectures on surgery. If to these thirty-nine years be added two years spent as demonstrator of anatomy in the Medical College of Ohio, and four years passed in the medical department of the Cincinnati College as professor of pathological anatomy, it will be perceived that my life as a public teacher extends over a period of forty-five years. During all this time it has been my good fortune to miss few lectures, either from sickness or any other cause. If my teaching has not always been of the best quality it has been as good as I knew how to make it. Whatever estimate may have been placed upon it by those who listened to it I can solemnly declare that it has always been earnest and conscientious, with an eye single to the interest of my pupils, the truths of medical science, and the honor and dignity of the profession. On no occasion have I entered the amphitheatre without due preparation. One of the great objects of my early professional life was to qualify myself for the occupation of a public teacher. This idea, which haunted me as I sat upon the hard benches of my alma mater, like the demon of Socrates, gave me no rest day or night. My first effort in this direction was made in this city, at the Franklin Institute, in the spring of 1830, the subject being general anatomy, a branch of study then little understood or cared for in this country. The effort, however, proved to be an abortive one. The novelty of the subject, my own inexperience, and the paucity of the students in the city at that time of the year were the causes of my failure. Finding practising and lecturing in so large a city to be up-hill work, I removed to Easton, in this State, whence, after two years and a half spent in earnest work, I went, in 1833, to Cincinnati as demonstrator of anatomy in the Medical College of Ohio. From this institution, after a service of two years, I was called to the chair of pathological anatomy in the Cincinnati College, in which I gave the first regular and systematic course of lectures on that most important branch of anatomy ever delivered in this country. In 1840 I was invited to the chair of surgery in the University of Louisville. In 1850 I became the successor of Dr. Valentine in the University of New York, but returned after the close of the session to the school in Kentucky. In 1856 I accepted the chair of surgery in my alma mater, unanimously tendered me by its honorable Board of Trustees.

"Having been thus actively engaged for so many years as a public teacher, it is not surprising that my pupils should be scattered over the country, while not a few of them are successfully practising in foreign climes. Upwards of five thousand diplomas bear my signature. Of the thirty-seven colleagues with whom I have at various times been associated, twenty-six have fallen by the wayside, for the most part ripe in years and full of honor, leaving eleven survivors, among others Williard Parker, Austin Flint, and John W. Draper, of New York; Benjamin Silliman, of New Haven, and our distinguished townsman, Joseph Pancoast, five men of whom any profession in any country might justly be proud. It has been said that youth is a blunder, manhood a struggle, and old age a regret. If this be true I have not realized it in my own person; nor need it be true of any one who is true to himself. Struggles of some kind or another are almost invariably the lot of every man who is not born with a silver spoon in his mouth. I certainly had mine, but they were the struggles of early life, and thank God for them, for they taught me patience and perseverance and self-reliance, and were powerful aids in developing character. These struggles did not discourage me. On the contrarv, I felt as Sheridan did when he made his maiden speech in the British House of Commons—that it was in me and would come out of me; or, as Erskine expressed it on a similar occasion, I felt that my children were tugging at my coat and urging me on to industry and perseverance that I might supply their necessities. A brave man never yields to despair. His motto is 'Perseverantia omnia vincit.' This has been my motto, and whatever success I have achieved is due to persistent effort and to a definite aim in life without any faltering or misgiving in regard to the final issue. I have never lost sight of the fact that what a man soweth he shall reap, or that 'if the spring show no blossoms, autumn will show no fruit.'

"Much has been said about the inspiration of genius. The greatest efforts that have ever been made at the forum, in the pulpit, or in the senate, in ancient or modern times, were the result of hard study and patient labor. Patrick Henry, William Pinckney, Rufus Choate, and others like these, never made a great argument or a great oratorical display without preparation, and the same is true of every profession and every pursuit. After fifty years of earnest work I still find myself in the harness; but although I have reached that age when most men, tired of the cares of life, seek repose in retirement and abandon themselves to the study of religion, the claims of friendship or the contemplation of philosophy, my conviction has always been that it is far better for a man to wear out than to rust out. Brain work, study, and persistent application has been a great comfort to me, as well as a great help; it has enhanced the enjoyment of daily life and added largely to the pleasures of the lecture room and of authorship; indeed it will always, I am sure, if wisely regulated, be conductive both to health and longevity. A man who abandons himself to a life of inactivity after having always been accustomed to work is, practically, dead.

"In taking a retrospect of my life I have no regrets. I console myself with the belief that I have not lived wholly in vain, and that while much remains undone that might and should have been done, it might be reasonable to suppose that at least some of the seed which I have sown have produced good fruit. It is not given to every man to be a Harvey, a Hunter, a Jenner, a Bichat, a Morton, a Paget or a Virchow. 'By the grace of God,' says St. Paul, 'I am what I am.' No man can rise superior to himself. What is fame? Is it a phantom or is it a reality? Alas! too often the former; too seldom the latter. Few medical works, however meritorious, outlive their authors, and no sooner does a teacher retire from the field of his labor than his pupils worship other gods. Happy, thrice happy, is he who in the evening of his life, as he reviews his past conduct, can say to himself, 'I have been true to my profession. I have been ambitious of its glory; I have done nothing to tarnish its escutcheon.' As I look back through the dark vista of half a century, what memories crowd upon my mind! Kingdoms have crumbled to pieces; new dynasties have sprung up; the world has been drenched in blood by contending armies; millions of human beings have been swept away by pestilence and famine; civilization, commerce, the arts and sciences, religion and education have found new homes; the uttermost parts of the globe have been explored by intrepid navigators and adventurous travellers; time and space have been annihilated by the telegraph, and the employment of steam and the application of machinery have changed the occupations of man and thrown upon us a surplus population, which the wisest statesmen know not how to dispose of. The art and science of medicine have been completely revolutionized and enriched to an extent which fifty years ago would have baffled the wildest conceptions. During these vast changes, so pregnant in beneficence to mankind, America has not been idle. If she had contributed nothing more to the stock of human happiness than anæsthetics, the world would owe her an everlasting debt of gratitude. The fanciful and mischievous speculations which characterized medicine in the days of my youth have been replaced by sober facts, founded upon more carefully conducted observations and more rational deductions. In preventive medicine a new field has been opened which, if properly explored and cultivated, as it seems destined to be, will add millions of years to the life of the human race. Oh! for a glance at the profession half a century hence, when man, enlightened and refined by education and redeemed from the thraldom of ignorance and superstition, shall reflect more perfectly than he now does the image of his Maker.

"I thank you, Mr. Chairman, and you gentlemen, who have honored me with your presence here this evening, for the patience and attention with which you have listened to my rambling remarks. Allow me before I take my seat, to wish you, one and all, prosperity and happiness, and to drink your health with a heart brimful of gratitude for the many favors that have been showered by my professional brethren upon me."

